# Supplementary material for: Characterizing cell-type spatial relationships across length scales in spatially resolved omics data
Source: Nat Commun. 2025 Jan 3;16:350. doi: 10.1038/s41467-024-55700-1 (PMC11699133; doi:10.1038/s41467-024-55700-1)
Supplement: Supplementary file 3 — Supplementary Software 1 [file 41467_2024_55700_MOESM3_ESM.zip › CRAWDAD/plots/spleen/3_spleen_pkhl_cd4_subset_trends_heatmap.pdf]

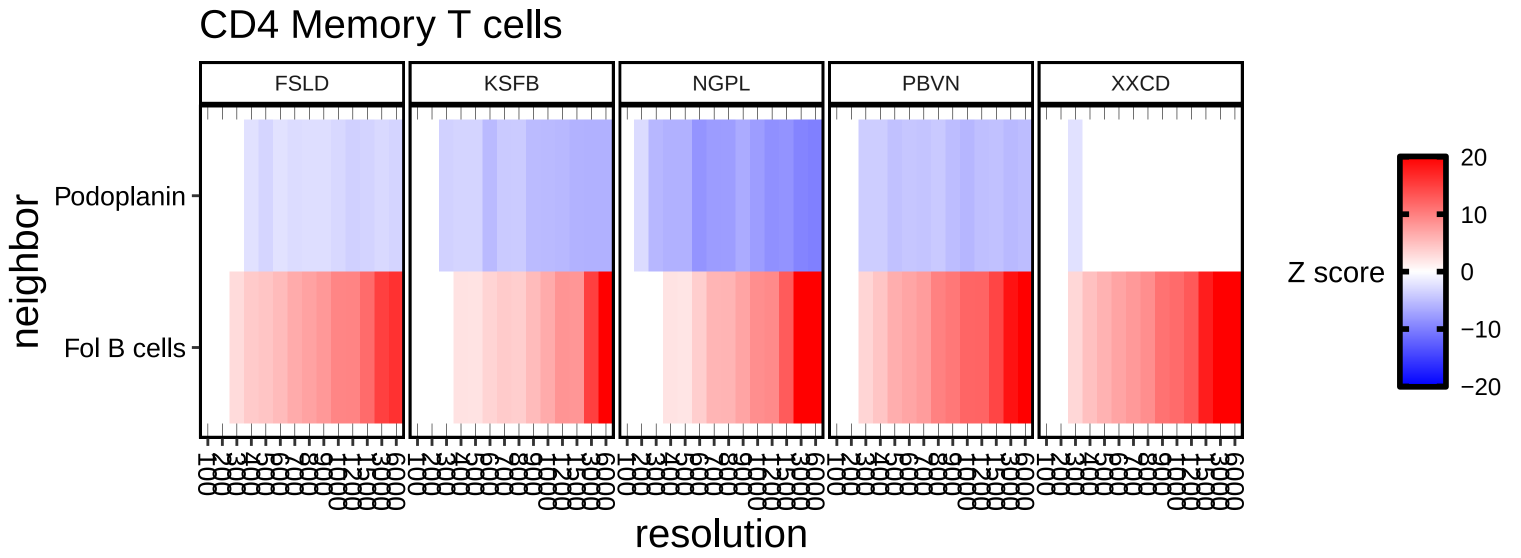

CD4 Memory T cells
FSLD
KSFB
NGPL
PBVN
XXCD
Podoplanin
Fol B cells
neighbor
resolution
Z score
20
10
0
−10
−20
